# The Genomic Standards Consortium

**DOI:** 10.1371/journal.pbio.1001088

**Published:** 2011-06-21

**Authors:** Dawn Field, Linda Amaral-Zettler, Guy Cochrane, James R. Cole, Peter Dawyndt, George M. Garrity, Jack Gilbert, Frank Oliver Glöckner, Lynette Hirschman, Ilene Karsch-Mizrachi, Hans-Peter Klenk, Rob Knight, Renzo Kottmann, Nikos Kyrpides, Folker Meyer, Inigo San Gil, Susanna-Assunta Sansone, Lynn M. Schriml, Peter Sterk, Tatiana Tatusova, David W. Ussery, Owen White, John Wooley

**Affiliations:** 1 Centre for Ecology & Hydrology, Maclean Building, Crowmarsh Gifford, Wallingford, Oxfordshire, United Kingdom; 2 The Josephine Bay Paul Center for Comparative Molecular Biology and Evolution, Marine Biological Laboratory, Woods Hole, Massachusetts, United States of America; 3 European Molecular Biology Laboratory (EMBL) Outstation, European Bioinformatics Institute (EBI), Wellcome Trust Genome Campus, Hinxton, Cambridge, United Kingdom; 4 Center for Microbial Ecology, Michigan State University, East Lansing, Michigan, United States of America; 5 Department of Applied Mathematics and Computer Science, Ghent University, Ghent, Belgium; 6 Department of Microbiology and Molecular Genetics, Michigan State University, East Lansing, Michigan, United States of America; 7 Argonne National Laboratory, Argonne, Illinois, United States of America; 8 Department of Ecology and Evolution, University of Chicago, Chicago, Illinois, United States of America; 9 Microbial Genomics Group, Max Planck Institute for Marine Microbiology and Jacobs University Bremen, Bremen, Germany; 10 Information Technology Center, The MITRE Corporation, Bedford, Massachusetts, United States of America; 11 National Center for Biotechnology Information, National Library of Medicine, National Institutes of Health, Bethesda, Maryland, United States of America; 12 DSMZ - German Collection of Microorganisms and Cell Cultures GmbH, Braunschweig, Germany; 13 Department of Chemistry and Biochemistry, University of Colorado, Boulder, Colorado, United States of America; 14 DOE Joint Genome Institute, Walnut Creek, California, United States of America; 15 Computation Institute, University of Chicago, Chicago, Illinois, United States of America; 16 LTER Network Office, Department of Biology, University of New Mexico, Albuquerque, New Mexico, United States of America; 17 University of Oxford, Oxford e-Research Centre, Oxford, United Kingdom; 18 Institute for Genome Sciences, University of Maryland School of Medicine, Baltimore, Maryland, United States of America; 19 Wellcome Trust Sanger Institute, Wellcome Trust Genome Campus, Hinxton, Cambridge, United Kingdom; 20 Center for Biological Sequence Analysis, The Technical University of Denmark, Lyngby, Denmark; 21 University of California San Diego, La Jolla, California, United States of America

## Abstract

A vast and rich body of information has grown up as a result of the world's enthusiasm for 'omics technologies. Finding ways to describe and make available this information that maximise its usefulness has become a major effort across the 'omics world. At the heart of this effort is the Genomic Standards Consortium (GSC), an open-membership organization that drives community-based standardization activities, Here we provide a short history of the GSC, provide an overview of its range of current activities, and make a call for the scientific community to join forces to improve the quality and quantity of contextual information about our public collections of genomes, metagenomes, and marker gene sequences.

## Introduction

We currently have thousands of genomes, hundreds of metagenomes, and tens of thousands of marker gene data sets in the public domain, and these numbers are rapidly increasing [1]. Next-generation sequencing technologies promise to further fill the public databases with a bounty of information unthinkable even a few years ago. Each data set represents an organism or community with a unique biological history, sampling location, environmental context, and set of biologically interesting traits. Hence, each of these data sets makes a unique contribution to the ongoing creation of our public online catalogue of life.

We are now witnessing the rapid democratization of access to sequencing capacity—an immense opportunity for the global community, if proper stewardship of these data keeps pace [2],[3]. This stewardship must include enriching public sequence databases with the biological context of these sequences (Box 1), which will in turn necessitate the adoption of a fresh attitude to reporting results, both in our papers and our submissions to the public databases. Large, well-contextualized genome, metagenome, and marker gene data sets (e.g., ribosomal gene surveys) provide ideal opportunities for comparison and contrasting using computational means to solve a wide range of questions in biology (including questions in medicine, physiology, developmental biology, biogeochemistry, evolution, ecology, etc.).

**Box 1. When the Cost of a Bacterial Genome Sequence Is Almost Nothing, That Organism's Contextual Information Is Increasingly Valuable**

Consider the scenario where a new *E. coli* sequence has been obtained from a futuristic handheld device (like a *Star Trek* tricorder) that generates the complete genome in seconds. While the genome sequence may only be slightly different from strains already in the public databases, the metadata associated with this bug is both unique and crucial. Where and when was the *E. coli* isolated? Was it transmitted as a food-borne pathogen? Did it hospitalize the patient from whom it was isolated? Was it part of a larger infectious outbreak? Knowledge that a pathogen was isolated from diseased patients or healthy controls will readily assist in intervention strategies derived from machine-readable data.

These data sets should be treated as part of a larger whole—a catalogue of life on earth—that will allow us to observe, as we sample in time and space, how life changes. A range of ongoing and proposed megasequencing projects also promise to make great inroads into this grand vision (i.e., the Genomic Encyclopedia of *Bacteria* and *Archaea* [GEBA] [4], Human Microbiome Project [HMP] [5], Microbial Earth Project [http://genome.jgi-psf.org/programs/bacteria-archaea/MEP/index.jsf], Earth Microbiome Project [6], Genomes 10K [7], Tara Oceans [http://oceans.taraexpeditions.org/], Malaspina [http://en.wikipedia.org/wiki/Malaspina_Expedition_2010], Sorcerer II Global Ocean Sampling expedition [8]).

How must we now change the way we think about these data sets to prepare to integrate and co-analyze these large suites of related and contrasting data? Clearly, these data must be stored in robust comprehensive electronic systems that link to specific environments, diseases, or physiological states such that these relationships are electronically retrievable. To achieve this goal we urgently need shared standards that are both easy to use and scientifically robust.

## The Genomic Standards Consortium

The GSC was established in late 2005 [9],[10] to tackle the challenge of working towards better descriptions of genomes and metagenomes through community-level, consensus-driven solutions. The GSC's mission is to work towards 1) the implementation of new genomic standards, 2) methods of capturing and exchanging the information captured in these standards (metadata, or contextual data) and 3) harmonization of information collection and analysis efforts across the wider genomics community.

The GSC fulfils this mission by holding face-to-face meetings, forming working groups, and building consensus products that can be widely used in this community. Thus far, the GSC has created a standard, the Minimum Information about any (x) Sequence (MIxS), that includes three minimum information checklists for describing genomes, metagenomes, and environmental marker sequences (MIGS/MIMS/MIMARKS) upon submission to the public databases and publication [11],[12]. MIxS requires core information on habitat, geolocation, and sequencing methodology as well as fields specific to data type and a range of optional environmental packages to capture core measurements defining a broad range of habitats, including water, soil, and host-associated habitats. The International Nucleotide Sequence Database Collaboration (INSDC; DDBJ/EMBL/GenBank) has created a GSC “keyword” (MIxS) to mark the richer entries complying with this standard.

Other working groups are dedicated to 1) the maintenance of an extensible markup language (GCDML) that provides a reference implementation of the MIxS checklists [13], 2) development of tools and software, 3) compliance and curation, and 4) biodiversity. Those requiring help complying with MIxS (curation support) should contact the compliance working group, and those requiring technical assistance in implementing/adopting these standards in software or database projects should contact the developer's working group (technical support). The developer and compliance groups work closely together, for example, to support compliance through a range of portals, including GOLD [1], MG-Rast [14], CAMERA [15], IMG/m [16], the RDP [17], SILVA [18], megx.net [19], and the ISA software suite [20]. The Biodiversity group works with communities to make sure that GSC standards evolve in harmony with standards for describing taxonomy and biodiversity.

The GSC has also stepped forward to create a journal designed to underpin the emerging field of standards development in the biological sciences [21]. The *Standards in Genomic Sciences* journal now serves as a formal voice for the GSC and supports the publication of standardized genome, metagenome, and pan-genome reports and other standards-supportive publications like Standard Operating Procedures (SOPs) [22] from the scientific community at large.

The GSC is now maturing into a hub for the coordination of large-scale projects. Two projects running under the GSC umbrella are the Microbial Earth Project, which calls for the coordinated sequencing of over 9,000 type strains (http://genome.jgi-psf.org/programs/bacteria-archaea/MEP/index.jsf), and the M5 project, which calls for the coordinated development of a next-generation computational infrastructure (http://gensc.org/gc_wiki/index.php/M5) [23].

The GSC also works closely with a range of related communities and helped drive the formation of the Environment Ontology [24], the Minimum Information for Biological and Biomedical Investigations (MIBBI) initiative [2], and most recently the BioSharing forum [3, 25].

## A Call for Participation and Adoption

The Internet has resulted in a Cambrian explosion of productivity and data sharing through the adoption of a huge stack of agreed-upon protocols (standards) that allow many devices and programs to communicate to the transformative benefit of the everyday user [26]. Enabling access to user-generated content is key to harnessing the resources of a distributed community: Flickr has over 5 billion photographs uploaded, and Wikipedia has over 3.5 million English articles as of this writing. Standards for organizing sequence data will be similarly needed as sequencing instruments themselves, especially as these instruments are more and more commoditized and owned by individuals rather than institutions.

The tagline of the GSC is “Innovation through Collaboration”. For any standard to create a lasting impact requires substantial input from the wider scientific community, including adoption and support. The GSC urges researchers interested in pushing the boundaries of genomic science through collaboration to join and contribute expertise to building the GSC roadmap for the future. Membership in the GSC and all working groups is currently defined by participation. The GSC has a Board and several standing committees in addition to its working groups. For more information on the GSC, please see http://gensc.org/.

## Conclusions

The GSC is working to become the authoritative working body in the area of genomics for the development and adoption of standards. We anticipate that the need for a collaborative body in which to build consensus at the community level and undertake large-scale projects will only increase with time, as in many ways the era of genomics is just beginning. In the future, sequence generation will only increase as access is further democratized. On one extreme, it will be like any other industrial commodity and will be outsourced into a global manufacturing marketplace. On the other, mid- to large-scale sequencing will be as locally accessible as a benchtop microscope or PCR machine is to a typical university researcher. Making these diverse streams of data accessible in a coherent framework will require new, standardized ways of describing, storing, and exchanging this information. The framework required to do this will involve acceptance of profound sociological and technological changes in how we do business in the genomic sciences.

## Abbreviations

GSC: Genomic Standards Consortium
MIxS: Minimum Information about any (x) Sequence
